# Canadian Conference on Medical Education

**Published:** 2020-04-18

**Authors:** Alan J. Neville, Anna Karwowska

**Affiliations:** 1 Department of Oncology, Associate Dean, Health Professional Education, Faculty of Health Sciences, McMaster University; 2 Education Association of Faculties of Medicine of Canada

Dear Colleagues,

Welcome to CCME 2020: a virtual CCME.

On March 11, 2020, the World Health Organization declared the COVID-19 outbreak a pandemic. The Government of British Columbia, directed all event organizers to cancel any gatherings over 250 people on March 12. The safety of CCME delegates and Canada’s health workforce is paramount and a high priority for the CCME partners, five national organizations including the Association of Faculties of Medicine of Canada, The Canadian Association of Medical Education, the College of Family Medicine of Canada, the Medical Council of Canada and the Royal College of Physicians and Surgeons of Canada. The Scientific Program Committee represents all five organizations and carefully prepares a conference program.

The program theme this year is “Weaving Humanism into the Fabric of Medical Education.” Although our medical schools facilitate the acquisition of scientific expertise, this alone cannot help patients deal with the loss of health and find some sort of meaning in their illness. We must graduate physicians who can and will listen to their patients and sufficiently understand their narratives of illness to act on their patients’ behalf. Any medical school that is serious about fostering a humanistic approach among its graduates must foster the development of supportive and collaborative learning environments that value mutual respect, professionalism and academic integrity. In these unprecedented times, with the stresses that are upon the entire healthcare system, this has never been more important.

We will leverage state of the art technology to bring you a virtual event with a condensed scientific program with Humanism as a focus. All original plenaries, as designed by the Scientific Program Committee, will be provided. These include the plenary sessions, the Education Innovation and Education Research symposia, the AFMC Learner Forum and AFMC Hot Topic. Abstracts for all posters, workshops and orals accepted to CCME 2020 will be published in the Canadian Medical Education Journal.

We look forward to seeing you, virtually, at the 2020 CCME.

Chers collègues,

Bienvenue à la CCEM 2020 : Une CCEM virtuelle.

Le 11 mars 2020, l'Organisation mondiale de la santé déclarait que l'épidémie de COVID-19 était une pandémie. Le 12 mars, le gouvernement de la Colombie-Britannique ordonnait à tous les organisateurs d’événements d'annuler tout rassemblement de plus de 250 personnes. La sécurité des délégués de la CCEM et du personnel de santé canadien est primordiale et constitue une priorité pour les cinq organismes nationaux partenaires de la CCEM, soit l'Association des facultés de médecine du Canada, l'Association canadienne pour l'éducation médicale, le Collège des médecins de famille du Canada, le Conseil médical du Canada et le Collège royal des médecins et chirurgiens du Canada. Représentant les cinq organismes, le Comité du programme scientifique prépare avec soin le programme de la Conférence.

Cette année, le programme de la Conférence a pour thème « Tisser l’humanisme dans la trame de l’éducation médicale ». Bien que nos facultés de médecine facilitent l'acquisition d'une expertise scientifique, celle-ci ne peut à elle seule aider les patients à faire face à la dégradation de leur santé et à trouver un certain sens à leur maladie. Nous devons former des médecins qui peuvent et veulent écouter leurs patients et qui comprennent suffisamment bien le récit de leur maladie pour agir au nom de leurs patients. Toute faculté de médecine qui souhaite sérieusement encourager une approche humaniste chez ses diplômés doit favoriser le développement de milieux d'apprentissage favorables et collaboratifs qui valorisent le respect mutuel, le professionnalisme et l'intégrité universitaire. En cette période sans précédent, avec les pressions qui s'exercent sur l'ensemble du système de santé, cela n'a jamais été aussi important.

Nous miserons sur une technologie virtuelle de pointe pour vous proposer une activité virtuelle et un programme scientifique condensé axé sur l'humanisme. Toutes les plénières originales, telles que conçues par le Comité du programme scientifique, seront offertes. Il s'agit notamment des séances plénières, des symposiums sur l'Innovation en éducation et la Recherche en éducation, du Forum des apprenants de l'AFMC et du sujet d’actualité de l'AFMC. Les résumés de toutes les affiches et présentations orales et de tous les ateliers acceptés dans le cadre de la CCEM 2020 seront publiés dans le *Canadian Medical Education Journa*l.

Nous nous réjouissons à l’idée de vous voir, virtuellement, à la CCEM 2020.


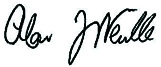
Dr. Alan J. Neville BMed Biol (Path), MBChB, MEd FRCP (Lond), FRCPC Professor, Department of Oncology Associate Dean, Health Professional Education Faculty of Health Sciences McMaster UniversityD^r^ Alan J. Neville BMed Biol (Path), MBChB, MEd FRCP (Lond), FRCPC Professeur, Département d’oncologie Doyen associé, Formation des professionnels de la santé Faculté des sciences de la santé Université McMaster
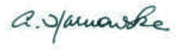
Anna Karwowska MDCM FRCPC Vice President, Education Association of Faculties of Medicine of CanadaAnna Karwowska MDCM FRCPC Vice-Présidente, Éducation L'Association des facultés de médecine du Canada

